# Omalizumab Updosing in Chronic Spontaneous Urticaria: an Overview of Real-World Evidence

**DOI:** 10.1007/s12016-020-08794-6

**Published:** 2020-05-16

**Authors:** Martin Metz, Zahava Vadasz, Emek Kocatürk, Ana M. Giménez-Arnau

**Affiliations:** 1 Department of Dermatology and Allergy, Charité – Universitätsmedizin Berlin, Berlin, Germany; 2 Division of Allergy and Clinical Immunology, Bnai-Zion Medical Center, Haifa, Israel; 3 Department of Dermatology, Koç University, Istanbul, Turkey; 4 Department of Dermatology, Hospital del Mar. IMIM, Universitat Autònoma de Barcelona, Barcelona, Spain

**Keywords:** Chronic spontaneous urticaria, Chronic idiopathic urticaria, Refractory urticaria, Omalizumab, Updosing, Real-world evidence

## Abstract

Chronic spontaneous urticaria (CSU) is defined as the spontaneous development of itchy hives and/or angioedema due to known or unknown causes that last for at least 6 weeks. At any given time, CSU is believed to affect 0.5–1% of the global population. Omalizumab (a recombinant, humanized anti-immunoglobulin-E antibody) is the only approved treatment for antihistamine refractory CSU. However, ~ 30% of patients remain symptomatic at licensed doses of omalizumab 150 mg and 300 mg, even after a treatment period of over 6 months. In the recent years, there have been several studies on updosing of the drug, suggesting that the individualized approach for urticaria treatment with omalizumab is useful. In this article, we provide an overview of these studies and the real-world data on omalizumab updosing as it became necessary to obtain complete CSU symptom control in a proportion of patients. Published observational studies (from June 2003 to October 2019) on the updosing of omalizumab in CSU were identified using PubMed and Ovid databases. Reports mainly show that updosing/dose adjustment evaluated with the assessment of disease activity (Urticaria Activity Score) and control (Urticaria Control Test) achieves better clinical response to omalizumab with a good safety profile in a pool of patients with CSU. These real-world data will provide an overview of updosing of omalizumab in CSU and aid in setting informed clinical practice treatment expectations.

## Introduction

Chronic spontaneous urticaria (CSU), a subgroup of chronic urticaria, is defined as the spontaneous daily, or almost daily, occurrence of itchy hives (wheals), angioedema, or both, lasting for 6 weeks or more, with no apparent external trigger [1]. CSU presents a major burden of disease for patients and society with a significantly diminished quality of life [2, 3]. The estimated lifetime point prevalence of CSU is approximately 0.5–1% and nearly 60% of patients with CSU continue to have the disease despite treatment with antihistamines at the licensed dose [4–6]. Approximately 33–67% of CSU cases have both hives and associated angioedema [7–9]. A recent investigation looking at differences in physician and patient reporting of angioedema showed that in 40% of inadequately controlled CSU patients angioedema are reported by both physicians and patients, but additionally, almost every third patient reported about occurrence of angioedema while the physician did not [10].

**Fig. 1:**
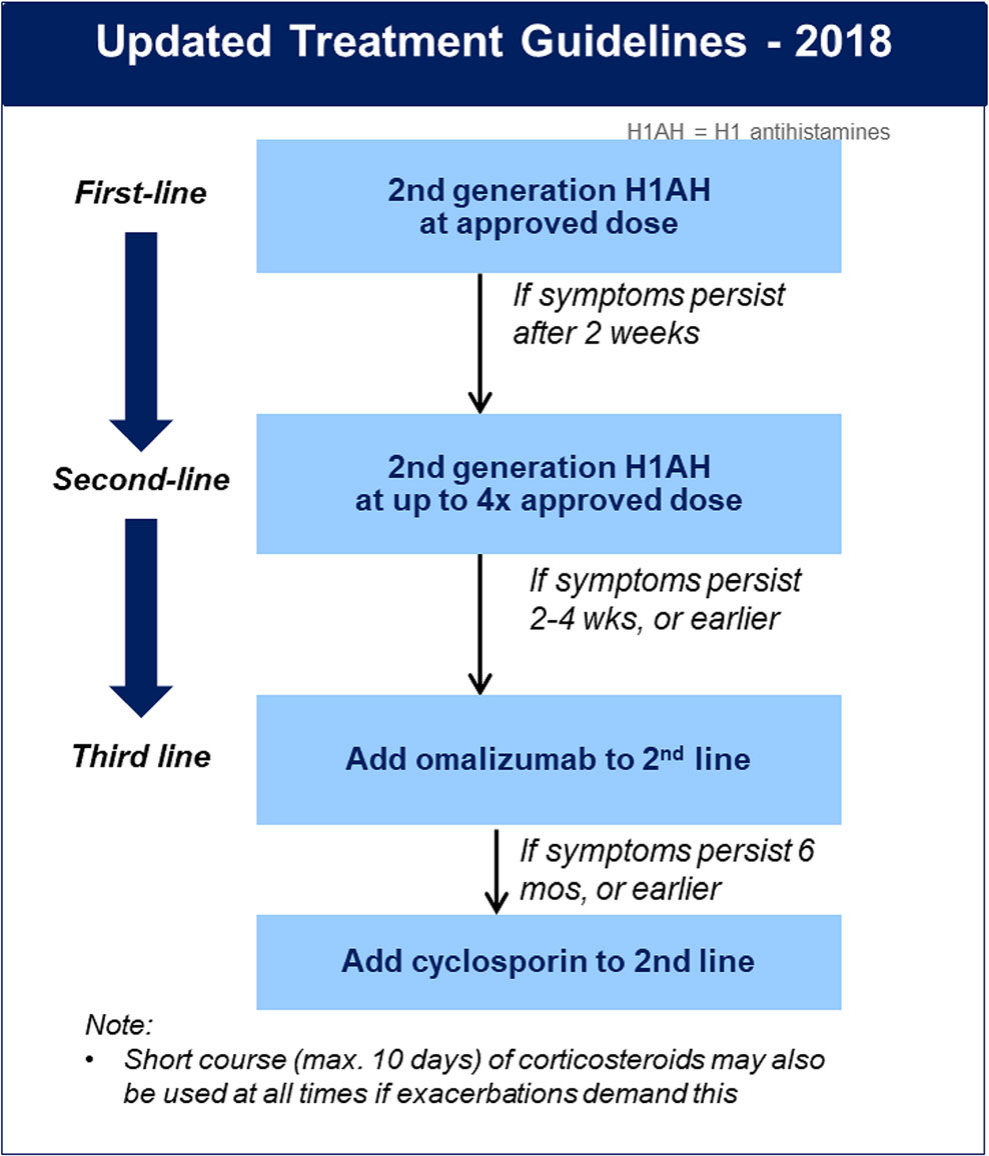
EAACI/GA^2^LEN/EDF/WAO International Guideline: recommended treatment algorithm for urticaria. Short course (maximum of 10 days) of corticosteroids may also be used at all times if exacerbations demand this. EAACI, European Academy of Allergology and Clinical Immunology; EDF, European Dermatology Forum; GA^2^LEN, Global Allergy and Asthma European Network; H1-AH, H1 antihistamine; WAO, World Allergy Organization

Much progress has been made recently to delineate the underlying mechanisms of CSU and the pathogenesis therein, and to use this understanding to develop better treatment options including immunoglobulin E (IgE)–targeted therapies, which show benefit in patients [11, 12]. The EAACI/GA^2^LEN/EDF/WAO guidelines recommend following a stepwise approach to treat urticaria (Fig. 1) [1]. Treatment with second-generation H1-antihistamines (H1-AHs) are the mainstay of symptomatic therapy of CSU, with treatment in licensed standard dosing as first-line, and updosing to up to four times the recommended standard dosing as the second-line treatment. The guideline recommended third-line therapy which is the use of omalizumab as third-line add-on therapy to H1-AHs, if an inadequate response to H1-AHs is observed after 2–4 weeks (or earlier if symptoms are intolerable). Patients who remain inadequately controlled with omalizumab after 6 months (or earlier if symptoms are intolerable) are recommended to receive add-on therapy with cyclosporin A as a fourth-line agent.

Omalizumab (a recombinant, humanized anti-IgE antibody) is an effective and well-tolerated treatment option for CSU and the first drug approved for use in patients with CSU who remain symptomatic despite H1-AH treatment. Omalizumab is shown to be safe and effective across randomized placebo-controlled trials [13–15] and several real-world studies [16–20], with a total patient exposure of 1,328,183 patient years (Novartis data on file, Dec 2019). Omalizumab binds IgE and rapidly reduces levels of free IgE by > 90%, resulting in a subsequent reduction of FcεRI, the high affinity IgE receptor, on blood basophils and mast cells in the skin [21]. Both of these mechanisms are thought to importantly contribute to the efficacy of omalizumab in urticaria [22]. Additional modes of action of omalizumab may exist in urticaria and further research will be necessary to fully clarify the potential of omalizumab in CSU [23]. The definition of response to treatment in CSU differs between clinical trials, real-world studies and daily practice of individual physicians globally [24], and, therefore, the response to treatment in CSU also depends upon how it is measured [25]. Commonly used categories for complete and well-controlled disease activity for CSU include Urticaria Activity Score (UAS) over 7 days (UAS7) of 0 and of ≤ 6 respectively. Omalizumab non-responders to treatment are generally considered to be those patients whose baseline UAS7 remains unchanged after treatment or who continue to present a UAS7 > 16 after six doses of omalizumab at 300 mg every 4 weeks. Partial responders are often defined by a reduction of the UAS7 by at least 30%, but by less than 90% or by patients showing a UAS7 > 6 but with an improvement in UAS7 as compared with baseline [25]. Using the Urticaria Control Test (UCT), the cutoff values for “controlled disease” is ≥ 12 which is often considered as a complete response to treatment (complete control = 16, controlled urticaria ≥ 12, no control < 12) [25]. As per the current EAACI/GA^2^LEN/EDF/WAO guideline, the third line of treatment is recommended for the partial and non-responders to updosed AH treatment [24]. The licensed dose of omalizumab is 300 mg in Europe and either 150 mg or 300 mg in the USA by subcutaneous injection every 4 weeks [26]. Some reports have shown examples of the optimization of omalizumab treatment in patients who show inadequate response by increasing the dose or decreasing the dosing intervals [27]; a more flexible treatment regimen rather than a constant regimen including shortening dosing interval or augmenting dose based on the patient’s symptoms is also likely to provide better symptom control [28, 29]. Although there is currently no algorithm for the individualized management of omalizumab treatment that is agreed on, treatment algorithms based on specific response profiles of patients refractory to AHs have been proposed to facilitate clinical management of omalizumab and enable clinicians to assess therapeutic strategy [2, 30, 31]. The aim of this article is to provide a summary of the published real-world evidences for the updosing of omalizumab in the treatment of CSU.

## Overview on Omalizumab

Omalizumab is a recombinant humanized IgG1 monoclonal antibody that binds to IgE-specific epitopes within the C3 (FcεRI binding) region of the IgE molecule, with low immunogenicity, that inhibits binding of IgE to FcεRI on the surface of mast cells and basophils [32–34]. On continued therapy, omalizumab is associated with downregulation of cell surface IgE receptors, further preventing IgE-mediated histamine release and inflammation.

## Omalizumab in the Treatment of Refractory CSU

Positive results from investigator-reported use of omalizumab for chronic urticaria provided an impetus for therapeutic treatment in CSU. Successful use of omalizumab in chronic urticaria began to appear in the literature as early as 2006 as case reports [35, 36]. The first controlled clinical trials in chronic spontaneous urticaria followed some years later and included the X-CUISITE trial [37] which showed a high efficacy of omalizumab in patients with CSU pre-selected for the presence of anti-thyroid peroxidase-specific IgE autoantibodies [37, 38] and the MYSTIQUE study [39] which showed improvement in CSU symptoms with omalizumab 300 mg and formed the basis of further investigations of efficacy and safety of omalizumab in CSU at this particular dose. Overall, there is much evidence for the efficacy and safety of omalizumab and treatment with 300 mg omalizumab every 4 weeks in patients with CSU [40]. Clinical trials for treatment of CSU with omalizumab generally provide data for up to 6 months; there are ongoing trials with omalizumab treatment for up to 1 year and for re-treatment effectiveness.

## Literature Search Methodology

A literature search was performed in PubMed and Ovid (from June 2003 to October 2019) for the identification of relevant studies on the updosing of omalizumab in CSU. The following search terms were used: “omalizumab” OR “omalizumab 450 mg” OR “omalizumab 600 mg” and “chronic spontaneous urticaria” OR “chronic idiopathic urticaria” OR “urticaria” OR “angioedema” AND “real-world evidence” AND/OR “updosing.” The search was also restricted to English language and studies in humans. Out of the total 87 hits obtained from the search, 17 publications with omalizumab updosing were identified. Of these, nine publications were finally included in the review. Publications on inter-dose updosing from 150 to 300 mg were excluded. Reference lists of the articles included were manually searched for additional relevant studies.

## Real-World Evidence of Omalizumab Treatment and Updosing in CSU

Several studies from real-life clinical practice have reported the safety and efficacy of omalizumab at approved dosing. In a systematic review of 84 publications, Bernstein et al. [41] reported the real-world effectiveness of omalizumab for treatment of CSU. The most common initiation dose was 300 mg (in 62.7% of patients), whereas 34.5% of patients received 150 mg and 2.8% received other regimens [41]. The most common dosing frequency (83.9%) was every 4 weeks. There is also much evidence on the long-term effectiveness and safety, including long-term omalizumab treatment from 1 to 5 years or longer [42–44]. Long-term outcomes in a real-world study from a large cohort of patients in the USA associated with omalizumab 300 mg use were improvements in disease control, disease course, and patient-reported symptoms that were observed at month 6 and continued beyond 24 months, particularly when AHs alone failed to control symptoms [45]. Across real-world settings, treatment with omalizumab was thus associated with improved clinical response and clinical improvement [46].

Despite our understanding of the long-term treatment of CSU, it is known that only a minority of patients have symptom control with standard-dosed H1-AH treatment. Updosing of second-generation AHs as recommended by the EAACI/GA^2^LEN/EDF/WAO urticaria guideline as second-line therapy can improve response, but many patients remain symptomatic. Although omalizumab in licensed dosing has been proven to be effective in H1-AH refractory patients, there are patients who do not achieve complete control. Here, we reviewed the published evidence for the use and safety of updosing omalizumab from the standard monthly 300 mg dose to higher doses of monthly 450 mg or 600 mg.

From the first report in 2014 by Fiorino et al. [27] in Italy to the most recent report in 2019 from Aghdam et al. [47] in the Netherlands, there have been a growing number of publications reporting real-world evidence for updosing of omalizumab in CSU (Table 1).

**Table 1:** List of all studies showing omalizumab updosing in chronic spontaneous urticaria

Author (year)	Country	Total number of patients, *N*	Updose of omalizumab
Fiorino et al. (2014)	Italy	2	450 mg/4 weeks
Metz et al. (2014)	Germany	30	300 mg/4 or 3 weeks; 450 mg/4 weeks
Barron et al. (2017)	Canada	149	450 mg/4 weeks; 600 mg/4 weeks
Asher et al. (2017)	Portugal	50	450 mg/4 weeks
Vadasz et al. (2017)	Israel	280	450 mg/4 weeks
Kocatürk et al. (2018)	Turkey	92	450 mg/4 weeks;
	Spain	80	600 mg/4 weeks
Curto-Barredo et al. (2018)	Spain	286	450 mg/4 weeks; 600 mg/4 weeks
Salman et al. (2019)	Turkey	72	450 mg/4 weeks
Aghdam et al. (2019)	Netherlands	166	450 mg/4 weeks; 600 mg/4 or 2 weeks

Fiorino et al. [27] reported a case for the long-term treatment of refractory severe CSU by omalizumab with a UAS7 > 38. The patient was not responding to omalizumab 300 mg for 9 months and achieved complete symptom control after updosing to the higher dose of 450 mg/month.

Metz et al. [17] from Germany published a retrospective analysis of 30 patients with CSU not sufficiently treated with updosed H1-AH. Complete symptom control and remission of their symptoms (reduction of 90% or more in UAS7) was observed in 25 of 30 (83%) patients, without the requirement of any other drugs after the first treatment with omalizumab. Among 25 patients who achieved complete remission, one patient was updosed from 150 to 300 mg/3 weeks. In a further 5 (of the 30) patients, the minimum effective dose of omalizumab was 300 mg every 3 or 4 weeks. Two of these patients showed significant improvement when updosed from 150 to 450 mg every 4 weeks. The findings suggest that patients who may not respond to omalizumab show complete response upon updosing.

Barron et al. [48] from Canada reported a prospective analysis of 149 patients refractory to treatment of CSU who were treated with omalizumab 150 mg every 4 weeks. The dose and dose interval of omalizumab was adjusted based on UAS and physician assessment. A total of 21 patients were updosed with omalizumab (two patients updosed to 375 mg, ten patients to 450 mg, and nine patients to 600 mg). From the total 149 patients observed, 52% achieved complete remission, 29% showed significant improvement, and 17% were refractory. However, the analysis did not consider the updosed patients separately.

Asher et al. [49] from Israel reported evidence from real-life experience on the beneficial effects of high-dose (450 mg monthly) omalizumab for 50 patients with severe, unresponsive CSU who did not respond to the standard 300 mg monthly omalizumab dose (Table 2). Response to the starting dose of omalizumab 300 mg was complete in 30 (60%), partial in 15 (30%), and failed in five (10%) patients. Patients who showed partial response/failed to respond to 6 or more omalizumab injections of 300 mg dose, improved with updosing to 450 mg; of the nine patients who were updosed to omalizumab 450 mg, only one patient failed to show a response to the higher dose; while 8 patients improved significantly. The mean time to response to the higher dose in these patients was 2.6 ± 0.9 months. A significant decrease in the UAS7 from 20 ± 9 to 7 ± 10 (*P* = 0.002) following omalizumab treatment was reported in the updosed patients (Table 2).

**Table 2:** Proportion of patients with chronic spontaneous urticaria achieving complete or partial response on updosing omalizumab from 300 to 450 or 600 mg every 4 weeks

Study	Updosed to 450 mg	Updosed to 600 mg	Efficacy parameter (UAS7/UCT)	Complete/partial response, %
Asher et al.	9		UAS7 ≤ 6	66.7 (*n* = 6)/22.2 (*n* = 2)
Kocatürk et al. Barcelona Istanbul	17	11	UCT ≥ 12 and UAS7 ≤ 6	64.3 (*n* = 18)
		11	UCT ≥ 12	72.7 (*n* = 8)
Vadasz et al.	78			64.1 (*n* = 50)
Curto-Barredo et al.	79		UAS7 ≤ 6	75.0 (*n* = 59)/25.0 (*n* = 20)
Salman et al.	13		UCT ≥ 12 and UAS7 ≤ 6	46.2 (*n* = 6)/23.1 (*n* = 3)
Aghdam et al.	11	33	UCT ≥ 12 and UAS7 ≤ 6	32.0 (*n* = 14)/30.0 (*n* = 13)

UAS7 Urticaria Activity Score over 7 days; UCT, Urticaria Control Test

Vadasz et al. [50] from Israel reported real-life experiences of 280 patients, where updosing omalizumab from 300 to 450 mg in 78 patients was significantly beneficial in 64.1% of patients (Table 2). The usefulness of increasing the dose above 300 mg was carefully assessed in this retrospective study for a large group of patients with refractory CSU. After 12 weeks of therapy, response was defined as well-controlled if improvement was > 80% from baseline (urticaria between 6 and 18 months; UAS7 12–18 points); 60–70%, fair response; 40–50%, weak response; and < 30%, failure. Weak responders and treatment failures had long-lasting urticaria, of 24–60 months, and UAS7 between 20 and 32 points. While fair responders continued only on AHs, weak responders required short courses of steroids. The dosage of omalizumab was increased from 300 to 450 mg after 3 months if the response to therapy was weak; further beneficial effect was seen in 64.1% (50/78) of patients with an increase to omalizumab 450 mg.

Kocatürk et al. [51], in a retrospective analysis of patients treated with omalizumab for CSU, proposed a protocol for updosing patients from two urticaria centers from Istanbul, Turkey, and Barcelona, Spain. From a total of 92 patients enrolled in Istanbul, 81 were treated with omalizumab 300 mg while 11 received omalizumab 600 mg. Patients who did not respond to omalizumab 300 mg achieved symptom control after directly being updosed to 600 mg. Response to updosing occurred in 8/11 patients (72.7%) who achieved a UCT score of ≥ 12 at week 12 of updosing (Table 2). From a total of 80 patients enrolled at the center in Barcelona, a stepwise dosing regimen was preferred, starting with 450 mg and updosed to 600 mg if there was no response. Urticaria control was achieved by 76.4% (13/17) of patients treated with omalizumab 450 mg and by 45.4% (5/11) of patients given 600 mg. The stepwise approach was thus recommended for patients with CSU starting from 450 mg and updosing to 600 mg who do not respond nor partially respond to 300 mg of omalizumab after 3–6 months of treatment. Updosing was required more often in patients with a body mass index (BMI) > 30 kg/m^2^ and with lower UCT scores at the baseline.

Curto-Barredo et al. [52] from Spain, in a recent observational multicentre study, showed that upon updosing of omalizumab in 80% of partial or non-responders (of 286 patients with CSU treated with omalizumab 300 mg every 4 weeks), 75% of patients achieved UAS7 ≤ 6 and disease control. Fifty-five percent of these patients were updosed to 450 mg every 4 weeks; 20% of patients who had received omalizumab 450 mg every 4 weeks were further updosed to 600 mg every 4 weeks. Patients with CSU were updosed with omalizumab if they were considered non-responder to standard-dosed omalizumab treatment. Here, non-response was defined as patients having a UAS7 > 6.

It was shown in a bivariate analysis that 41% of the high-dose (450–600 mg/4 weeks) versus 21% of licensed-dose responders (300 mg/4 weeks) frequently used cyclosporin A immediately before start of the anti-IgE therapy. In the multivariate analysis, patients with BMI ≥ 30 kg/m^2^ were associated with updosed omalizumab (odds ratio 1.14; *P* = 0.004) and a predicted likelihood of greater success with omalizumab treatment in these patients. The variables included in the analysis were baseline once-daily UAS7, inducible urticaria, angioedema, sex, age, BMI, total immunoglobulin (Ig) E, d-dimer, and previous immunosuppressive treatments. Likewise, patients aged > 57 years old showed a significant association with omalizumab updosing (odds ratio 1.038; *P* = 0.013).

Salman et al. [53] reported the effectiveness and safety of omalizumab 450 mg in a retrospective cohort study of 72 patients treated with omalizumab 300 mg and 450 mg. Of 13 patients with CSU who were unresponsive to omalizumab 300 mg and updosed to 450 mg, six had complete response and three had good disease control with a mean UAS7 that decreased from 18.6 to 5.1 and a mean UCT score that increased from 8.6 to 12.0. A partial response to omalizumab updosing was noted in 2 patients, while 2 patients were non-responders. No adverse events were reported during the entire study period. It was of interest to note that lower baseline total IgE levels were used as a predictor of non-response to omalizumab and the need for higher doses. Patients were grouped according to baseline IgE levels as high or low; updosed patients generally had lower IgE levels.

Aghdam et al. [47] recently demonstrated that updosing omalizumab from 300 to 450 mg or 600 mg every 4 weeks (in 44 of 166 patients) resulted in a clinical benefit in 61% of these patients who were not responsive to the initial dose of 300 mg. If the treatment response after three doses of omalizumab 600 mg every 4 weeks was insufficient, the subsequent treatment interval was shortened to 2 weeks. Omalizumab was discontinued if two consecutive doses of 600 mg at 2-week intervals yielded an insufficient response. The effects of updosing were examined by comparing disease activity prior to starting omalizumab treatment and at the end of the high-dose treatment. UAS7 at the end of the high-dose treatment was improved compared with UAS7 before dose increase (median of 20.0 vs 4.3, respectively). The additional effect of updosing was shown by comparing the effect of standard dose with the high dose, with improved clinical treatment after updosing observed in 61% of patients; 32% had a complete response and 30% had a partial response. Patient and treatment characteristics did not differ significantly between patients treated with standard-dosed and updosed omalizumab.

## Discussion

The objective of this review article was to summarize the published real-world evidence on the effects of updosing omalizumab for the clinical management of patients with CSU who do not respond to the licensed dose or the prescribed initial dose of omalizumab. The results presented in this review article have been extracted from several published reports, and provide comprehensive evidence that omalizumab updosing can result in improvements in UAS7, UCT, and quality of life scores in patients who were not responding sufficiently to standard dose of omalizumab. This report also highlights evidence suggesting that omalizumab updosing is associated with complete response rates in up to 60% of patients with refractory CSU. High dose of omalizumab is shown to be beneficial in patients with CSU who either failed or had partial response to the standard 300 mg dose of omalizumab treatment.

The reports assessed in this article suggest that updosing omalizumab in patients with no response at any time during dosing intervals after three doses or with partial response after 6 months of treatment at the maximum licensed dose of 300 mg can be considered. There is also increasing evidence that updosing to omalizumab 600 mg either directly or with a stepwise approach starting from 450 mg and then updosing to 600 mg if there is no response after omalizumab treatment results in better disease control.

In addition, there is some evidence that other factors may be seen to influence the updosing of omalizumab. Better clinical response with regard to disease severity for instance could be achieved by updosing omalizumab along with adjustments to the frequency of omalizumab treatment. Patients with higher BMI are more likely to require higher doses of the drug for treatment and achieve greater success with updosing with omalizumab. Also, patients previously treated with cyclosporin A and older patients (> 57 years of age) who may be non-responders to the standard dose can be expected to have greater success with updosing omalizumab [52]. Patients with lower IgE levels are more likely to be non-responders to omalizumab and therefore required updosing more often than the patients without [53]. Overall, it was observed that updosing in suboptimal responders was safe and effective. Patients receiving updosed omalizumab in general had higher BMI, lower pre-omalizumab UCT scores, and lower IgE levels. There was also no particular association with gender, associated angioedema, baseline UAS7 scores or inducible urticaria, and increased treatment success rate with updosing.

Omalizumab has a well-established safety profile at higher doses in severe allergic asthma and has been extensively used to treat adult and pediatric populations in clinical trials and in real-world practice [54, 55]. The benefits of omalizumab updosing reported in the real-world treatment of CSU exceed those reported in clinical trials, while the real-world safety profile is similar to that reported in clinical trials. The real-world treatment setting offers benefit to a heterogeneous population of patients affected by CSU and real-life data on their safety and efficacy profiles. The experiences on the use of doses higher than the licensed dose of omalizumab 300 mg provide support to recommend these higher doses for patients who are partial/non-responders and refractory to treatment. One of the current limitations with omalizumab treatment in patients with CSU is the fixed dosing schedule without options to adapt the therapy to individual patients. Dose optimization with omalizumab with the potential for updosing in patients not achieving complete remission is shown to provide clinical benefit in a considerable number of patients. These real-world data will provide an overview of updosing of omalizumab in CSU and aid in setting informed clinical practice treatment expectations.
